# Long-term electrical stimulation at ear and electro-acupuncture at ST36-ST37 attenuated COX-2 in the CA1 of hippocampus in kainic acid-induced epileptic seizure rats

**DOI:** 10.1038/s41598-017-00601-1

**Published:** 2017-03-28

**Authors:** En-Tzu Liao, Nou-Ying Tang, Yi-Wen Lin, Ching Liang Hsieh

**Affiliations:** 1 Graduate Institute of Chinese Medicine, College of Chinese Medicine, China Medical University, Taichung, 40402 Taiwan; 2 School of Chinese Medicine, College of Chinese Medicine, China Medical University, Taichung, 40402 Taiwan; 3 School of Post-Baccalaureate Chinese Medicine, College of Chinese Medicine, China Medical University, Taichung, 40402 Taiwan; 4 Graduate Institute of Acupuncture Science, College of Chinese Medicine, China Medical University, Taichung, 40402 Taiwan; 5 Research Center for Chinese Medicine and Acupuncture, China Medical University, Taichung, 40402 Taiwan; 6 Graduate Institute of Integrated Medicine, College of Chinese Medicine, China Medical University, Taichung, 40402 Taiwan; 7 Department of Chinese Medicine, China Medical University Hospital, Taichung, 40447 Taiwan

## Abstract

Seizures produce brain inflammation, which in turn enhances neuronal excitability. Therefore, anti-inflammation has become a therapeutic strategy for antiepileptic treatment. Cycloxygenase-2 (COX-2) plays a critical role in postseizure brain inflammation and neuronal hyperexcitability. Our previous studies have shown that both electrical stimulation (ES) at the ear and electro-acupuncture (EA) at the Zusanli and Shangjuxu acupoints (ST36–ST37) for 6 weeks can reduce mossy fiber sprouting, spike population, and high-frequency hippocampal oscillations in kainic acid (KA)-induced epileptic seizure rats. This study further investigated the effect of long-term ear ES and EA at ST36–ST37 on the inflammatory response in KA-induced epileptic seizure rats. Both the COX-2 levels in the hippocampus and the number of COX-2 immunoreactive cells in the hippocampal CA1 region were increased after KA-induced epileptic seizures, and these were reduced through the 6-week application of ear ES or EA at ST36–ST37. Thus, long-term ear ES or long-term EA at ST36–ST37 have an anti-inflammatory effect, suggesting that they are beneficial for the treatment of epileptic seizures.

## Introduction

Epilepsy is a chronic brain disorder that results in the sporadic occurrence of spontaneous seizures. Brain-damaging events such as encephalitis, traumatic brain injury, and stroke can induce inflammation in the central nervous system (CNS). This inflammation contributes to enhanced neuronal excitability and the onset of epilepsy^1^. Inflammation plays a critical role in epileptogenesis and ictogenesis, and epileptic seizures enhance the production of inflammatory mediators such as interleukin-1ß (a proinflammatory cytokine) and prostaglandins; these mediators stimulate the inflammatory process and enhance neuronal excitability^2^. Thus, brain inflammation can enhance neuronal excitability and seizure production, and seizures can cause inflammation. Anti-inflammation maybe a therapeutic strategy for the treatment of epilepsy^3^.

Cycloxygenase-2 (COX-2) enzyme levels rapidly increase during seizures, and COX-2 plays a critical proinflammatory role in postseizure brain inflammation and neuronal hyperexcitability. Accordingly, pretreatment with COX-2 inhibitors can reduce seizure severity^4^. Astrocytes play a crucial role in the initiation of seizures through the release of glutamate from extrasynaptic sources; this initiates the occurrence of paroxysmal depolarization shifts^5^.

The calcium-binding protein S100-B is mainly synthetized in and secreted from astrocytes. S100 combines with receptors for advanced glycation end-product (RAGE) in the extracellular matrix and plays a critical role in the glial modulation of neuronal synaptic plasticity^6^. The serum levels of S100-B are increased in children with temporal lobe epilepsy^7^. High RAGE expression is observed in inflammatory lesions, and the blocking of RAGE delays the development of an inflammatory response^8^.

The metabotropic glutamate receptor subtype-3 (mGluR3) is upregulated in the reactive astrocytes of kainic acid (KA)-treated mice^9^, and mGluR3 expression is increased in the mesial temporal lobe of an epileptic rat model; these represent the changes in glial and neuronal communication^10^.

Monocyte chemoattractant protein-1 (MCP-1) is a chemokine that plays a pivotal role in the regulation of migration and infiltration of monocytes/macrophages in response to inflammation^11^. The levels of MCP-1 and its CC chemokine receptor-2 (CCR2) are increased in intractable epilepsy patients with hippocampal sclerosis; this finding is correlated with the disease duration^12^.

Auricular acupuncture can be used to treat suspected epilepsy cases; this treatment can increase parasympathetic activity, which then activates the solitary tract nucleus and interferes with the synchronization of electroencephalograms (EEGs) from the subcortex; it also simultaneously activates the cholinergic anti-inflammatory pathway for controlling inflammation^13^. The signal induced by auricular acupuncture projects to the solitary tract nucleus through the auricular branch of the vagus nerve^14^. The solitary tract nucleus mediates the anticonvulsive effect of vagus nerve stimulation, and the stimulation of the solitary tract nucleus can affect the development of seizures in cats^15, 16^. Peripheral muscarinic receptors mediate the anti-inflammatory effects of auricular acupuncture^17^. Electro-acupuncture (EA) at the bilateral Zusanli acupoints (ST36) produces an antiepileptic effect and increases the expression of GDA67 (glutamic acid decarboxylase) mRNA, which is a marker for γ-amino-butyric acid (GABA)-sensitive neurons in the dentate gyrus of lithium–pilocarpine-induced epilepsy rats^18^. EA at ST36 can increase the production of c-fos (a cellular marker of neural activity) in the solitary tract nucleus^19^. Our previous studies have shown that both 2-Hz electrical stimulation (ES) at the ear and 2-Hz EA at Zusanli and Shangjuxu acupoints (ST36–ST37) for 6 weeks can reduce mossy fiber sprouting as well as spike population and high-frequency hippocampal oscillations, which are both biomarkers of epileptogenesis in KA-induced epileptic seizure rats^20, 21^ and which can reduce epileptogenesis. We hypothesized that both ear ES and EA at ST36–ST37 could produce an anti-inflammatory effect. Therefore, the present study used Western blot and immunohistochemical (IHC) staining analyses to investigate the effects of long-term ear ES and long-term EA at ST36–ST37 on COX-2, glial fibrillary acidic protein (GFAP), S100-B, RAGE, mGluR3, MCP-1, and CCR2 in KA-induced epileptic seizure rats.

## Materials and Methods

### Animals

Male Sprague–Dawley (SD) rats weighing 200–300 g were purchased (BioLASCO Taiwan Co., Ltd) and raised in the animal center of China Medical University (CMU). A 12–12-h light–dark cycle was maintained, and the room temperature was controlled at 25 °C. Adequate food and water were provided. The Animal Care and Use Committee of CMU approved the use of these animals. In addition, all procedures were performed according to the *Guide for the Use of Laboratory Animals* (National Academy Press).

## Epileptic seizure rat model

### Preparation of electrodes

Thirty SD rats were placed in a stereotactic apparatus in a prone position under isoflurane (Aerrane, Canada) anesthesia administered through a vaporizing system (MATRX VIP 3000, Midmark, USA). The methods used in this study were similar to those described in our previous study^21^. In summary, the rats’ scalp hair was cut using surgical scissors, and a surgical knife was used to incise the scalp at the midline to expose the skull. Stainless steel screw electrodes, which were placed on the dura above the bilateral sensorimotor cortices, served as the recording electrodes. A reference electrode was placed at the frontal sinus for EEG recordings. Bipolar electrical wires were passed through the subcutaneous tissue and around the neck muscles for electromyogram (EMG) recordings. The electrodes were plugged into a conductor, which was affixed to the skull with dental acrylic cement. These electrodes were then connected to EEG- and EMG-monitoring machines (MPIOOWSW, BIOPAC Systems, Inc., CA, USA). Epileptic seizure behaviors were confirmed using a video-recording epileptic behavioral analysis system (SeizureScan, Clever Sys., Inc., Virginia, USA), and EEG and EMG findings were recorded during a conscious and free-moving state for at least 4 days after electrode implantation. On EEG recordings, intraperitoneal injection (i.p.) of KA (12 mg/kg) was observed to mainly induce epileptic seizure behaviors, namely wet-dog behavior, facial myoclonia, and paw tremors, and epileptiform discharges. Epileptic seizure behaviors were observed on EEG and EMG recordings from 15 min before to 3 h after KA injection.

### Grouping

In the present study, the treatment groups included only those rats who exhibited wet-dog shake counts of >250 from the start to 3 h after KA injection; the rats in the normal group did not receive KA injection. The rats were randomly divided into five experimental groups, and each group contained six rats as follows: 1) normal group, in which the rats were peritoneally injected with phosphate buffer solution (PBS); 2) KA group, in which the rats were injected with KA (12 mg/kg i.p.); 3) sham group, in which two stainless steel acupuncture needles were inserted into the subcutaneous layer at ST36–ST37, and the needles were connected to electric stimulator without electric charge. Electric stimulation was applied for 3 days per week at 20 min/day for 6 weeks, starting from the next day after KA injection; 4) auricular group, in which the rats received 2 Hz ES (using clip electrodes with the anode placed at the ear apex and cathode at the ear lobe; stimulus frequency, 2 Hz; stimulus intensity, visual ear twitch; stimulus duration, 20 min/day with each ear receiving the stimulus for 10 min) for 3 days per week for 6 weeks continuously, starting from the next day after KA injection; and 5) Zusanli group, in which the rats received 2 Hz EA at ST36–ST37 (through the insertion of two stainless steel acupuncture needles into the muscle layer, with the anode placed at ST36 and cathode at ST37; stimulation intensity, visible muscle twitch) for 3 days per week for 6 weeks continuously, starting from the next day after KA injection. All the rats were sacrificed at 6 weeks, and the rat brains were removed after KA or PBS injection. The left hippocampus was used for immunohistochemistry (IHC) staining and the right hippocampus for Western blot studies.

### Western blot analysis

Right hippocampi were immediately excised for protein extraction. Total protein was prepared by homogenizing the hippocampi for 1 h at 4 °C in a lysis buffer containing 20 mmol/L of imidazole-HCl (pH 6.8), 100 mmol/L of KCl, 2 mmol/L of MgCl_2_, 20 mmol/L of ethyleneglycoltetraacetic acid (pH 7.0), 300 mmol/L of sucrose, 1 mmol/L of NaF, 1 mmol/L of sodium vanadate, 1 mmol/L of sodium molybdate, 0.2% Triton X-100, and a proteinase inhibitor cocktail. From each sample, 30 μg protein was extracted and analyzed through a bicinchoninic acid protein assay. The protein was subjected to 10–15% sodium dodecylsulfate–Tris–glycine gel electrophoresis and was transferred to a nitrocellulose membrane. The membrane was blocked with 5% nonfat milk in a TBST buffer (10 mmol/L of Tris, pH 7.5; 100 mmol/L of NaCl; and 0.1% Tween 20) and was incubated overnight at 4 °C with the primary antibodies in TBST containing bovine serum albumin. Peroxidase-conjugated antibody (1:500) was used as the secondary antibody. The membrane was assessed using the ECL-Plus protein detection kit.

### IHC staining

The rats were anesthetized with chloral hydrate (400 mg/kg, i.p.) and then intracardially perfused with saline. The brains were removed and postfixed in the same fixative overnight at 4 °C. After briefly washing with PBS, the brains were transferred to a 30% sucrose solution in 0.01 M PBS for cryoprotection, and coronal sections containing the hippocampal area were cut into 16-μm-thick slices through cryosectioning. The sections were preincubated for 10 min at room temperature with 10% normal goat serum in PBS to avoid nonspecific binding. The sections were incubated overnight at 4 °C in PBS containing the primary antibodies to COX-2 (Cell Signaling, USA; 1:1000), GFAP (Calbiochem, Germany; 1:500), S100-B (1:1000; Novus Biologicals, USA), RAGE (Abcam, UK; 1:1000), mGluR3 (1:1000; Abcam, UK), MCP-1 (1:1000; Abcam, UK), CCR2 (1:1000; Abcam, UK), and actin (1:1000; Millipore, USA). The sections were subsequently incubated with the biotinylated-conjugated secondary antibody (diluted at 1:200; Vector, Burlingame, CA 94010, USA) for 10 min at room temperature, followed by incubation with the avidin–horseradish peroxidase complex (ABC kit, Genemed, USA). The sections were finally visualized using 3,3′-diaminobenzidine as the chromogen. During the incubation steps, the sections were washed with PBS three times for 10 min per cycle. The stained hippocampus slices were sealed under the coverslips, and then examined for the presence of immune-positive hippocampal neurons using a microscope (Olympus, BX-51, Japan) with a 40× numerical aperture (NA = 1.4) objective. Where applicable, the immune-positive signals were quantified with NIH ImageJ software (Bethesda, MD, USA).

### Double immunofluorescence analysis

The brain sections were blocked for 10 min in PBS containing 10% bovine serum albumin (Sigma, USA) and then incubated overnight at 4 °C with the primary antibodies [1:1000 rabbit polyclonal S-100B (Novus bio, USA) and 1:500 mouse monoclonal GFAP (Calbiochem, USA)]. Subsequently, the sections were incubated with the secondary antibodies [1:800 Alexa Fluor 488-conjugated donkey anti-rabbit and 1:800 Alexa Fluor 594-conjugated goat (Jackson ImmunoResearch Lab. Inc., USA)] for 1 h at room temperature. Each of the aforementioned steps was followed by three 3-min rinses in 0.01% Tween 20/PBS. At the end of the procedure, the sections were coverslipped using a mounting medium (Sigma, USA) containing 4′,6-diamidino-2-phenylindole to counterstain the DNA in the nuclei and were then dried overnight. Confocal images were captured using a laser-scanning confocal microscope (Leica TCS SP2, Germany).

### Statistical analysis

All data are presented as mean ± standard deviation. Statistical significance among the normal, KA, sham, auricular, and Zusanli groups was analyzed through one-way ANOVA, followed by Tukey’s post hoc test. A p value of <0.05 was considered statistically significant.

## Results

### KA-induced epileptic seizures in rats

**Figure 1:**
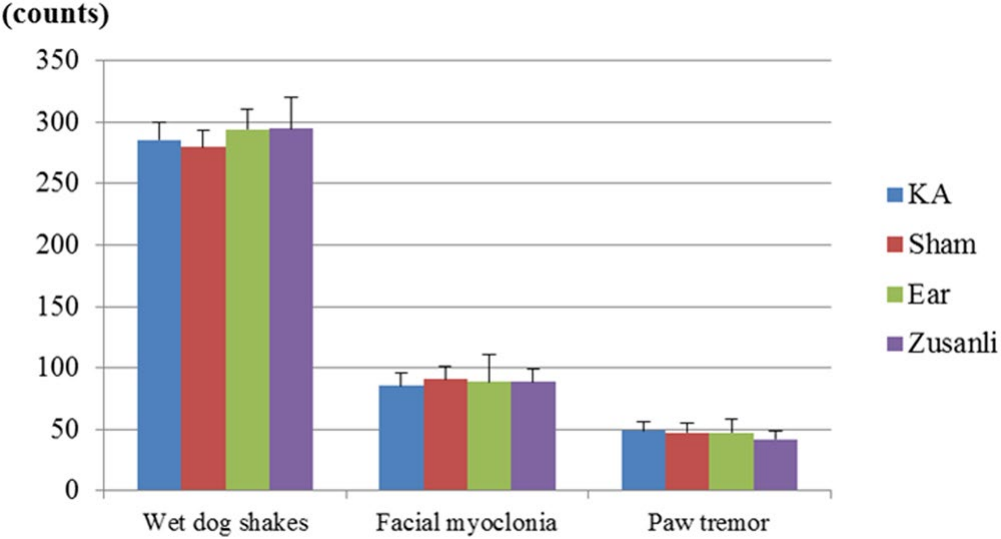
Kainic acid (KA)-induced epileptic seizures in rats. The counts of KA-induced wet-dog shakes, facial myoclonia, and paw tremors were similar among the KA group (KA), sham group (Sham), auricular group (Ear), and Zusanli group (Zusanli).

Among the KA-treated rat groups, the counts for wet-dog shakes were 285.3 ± 14.0 in the KA group, 275.8 ± 16.3 in the sham group, 294.5 ± 25.3 in the auricular group, and 294.7 ± 24.8 in the Zusanli group. However, no significant differences were observed among these groups (all p > 0.05; Fig. 1).

Among the KA-treated rat groups, the counts for paw tremors were 48.7 ± 7.7 in the KA group, 47.2 ± 11.1 in the sham group, 47.2 ± 6.9 in the auricular group, and 41.8 ± 10.6 in the Zusanli group. However, no significant differences were observed among these groups (all p > 0.05; Fig. 1).

Among the KA-treated rat groups, the counts for facial myoclonia were 85.5 ± 10.5 in the KA group, 90.7 ± 22.0 in the sham group, 88.3 ± 10.1 in the auricular group, and 88.5 ± 14.6 in the Zusanli group. However, no significant differences were observed among these groups (all p > 0.05; Fig. 1).

Thus, the KA, sham, auricular, and Zusanli groups showed similar baseline values.

### Effect of 6-week ear ES and EA at ST36-ST37 on the levels of COX-2, GFAP, S100-B, RAGE, mGluR3, MCP-1, and CCR2 in KA-induced epileptic seizure rats

#### Western blot analysis findings

**Figure 2:**
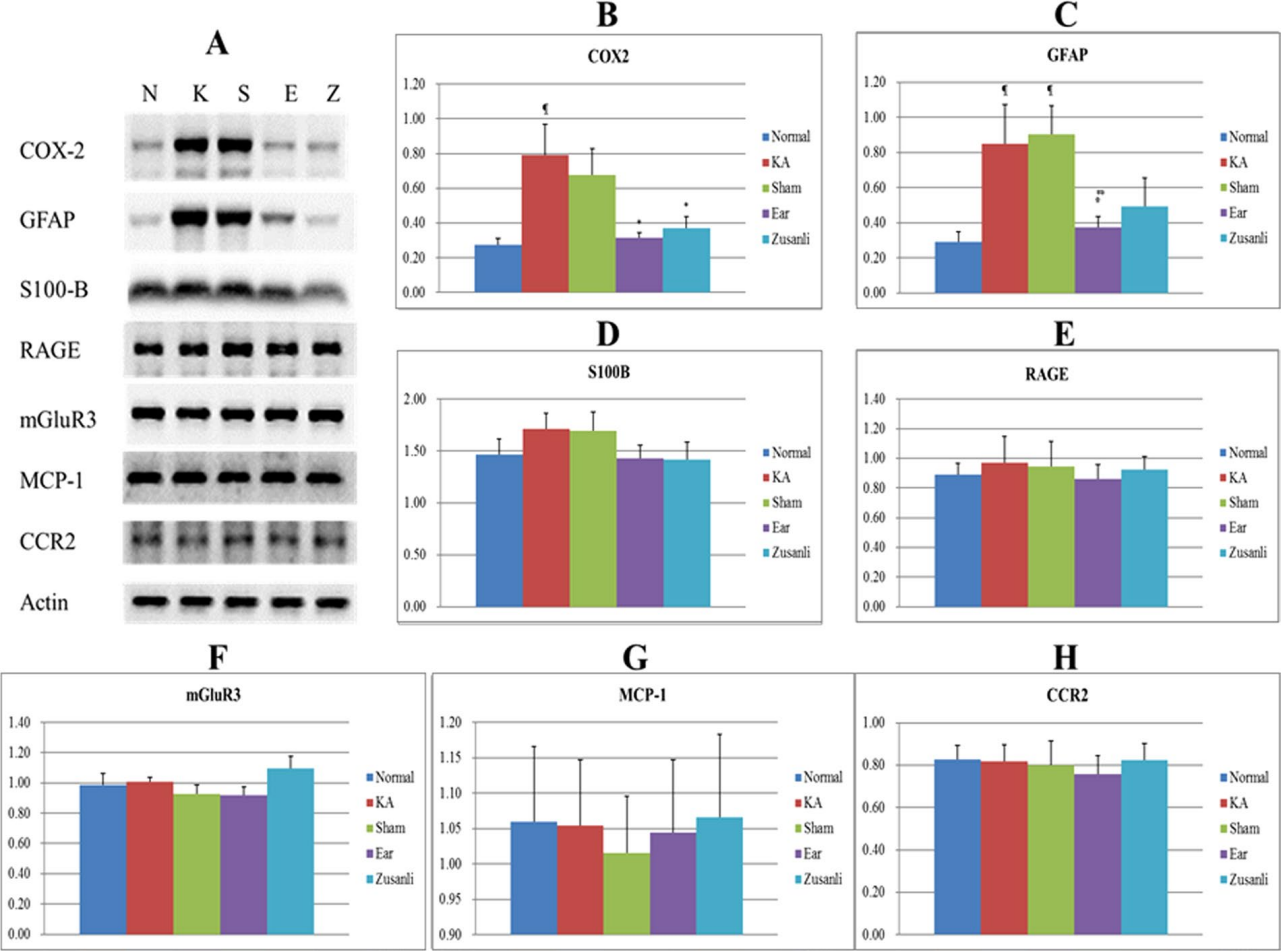
Western blot findings for the effects of electrical stimulation at the ear and electro-acupuncture at ST36–ST37 on the levels of COX-2, GFAP, S-100B, RAGE, mGluR3, MCP-1, and CCR2 in kainic acid (KA)-induced epileptic seizure rats. The levels of COX-2 increased in the KA group (K) and sham group (S), and decreased in the auricular group (E) and Zusanli group (Z) (**A** and **B**). The levels of GFAP increased in the KA group and sham group and decreased in the auricular group and Zusanli group (**A** and **C**). The levels of S100-B (**A** and **D**), RAGE (**A** and **E**), mGluR3 (**A** and **F**), MCP-1 (**A** and **G**), and CCR2 (**A** and **H**) among the normal group (N) and the KA, sham, auricular, and Zusanli groups. ^¶^p < 0.05 compared with the value of N; *p < 0.05 compared with the value of K; ^#^p < 0.05 compared with the value of S; n = 6.

The hippocampal COX-levels in the KA group were 79% ± 18%, which were higher than those in the normal group (27% ± 4%), auricular group (31% ± 3%), and Zusanli group (37% ± 7%; all p < 0.05; Fig. 2A and B; n = 6). The COX-2 levels in the sham group (68% ± 15%) were similar to those in the KA group (p > 0.05; Fig. 2A and B; n = 6).

The hippocampal GFAP levels in the KA (85% ± 22%) and sham (91% ± 16%) groups were higher than those in the normal (29% ± 6%) and auricular (37% ± 6%) groups (all p < 0.05; Fig. 2A and C; n = 6). The GFAP levels in the Zusanli group were 49% ± 16%, which were similar to those in the KA and sham groups (p > 0.05; Fig. 2A and C; n = 6).

The hippocampal S100-B levels were 147% ± 15% in the normal group, 171% ± 15% in the KA group, 170% ± 18% in the sham group, 143% ± 12% in the auricular group, and 141% ± 17% in the Zusanli group. However, no significant differences were observed among these groups (all p > 0.05; Fig. 2A and D; n = 6).

The hippocampal RAGE levels were 89% ± 7% in the normal group, 97% ± 18% in the KA group, 95% ± 17% in the sham group, 86% ± 10% in the auricular group, and 93% ± 9% in the Zusanli group. However, no significant differences were observed among these groups (all p > 0.05; Fig. 2A and E; n = 6).

The hippocampal mGluR3 levels were 99% ± 7% in the normal group, 101% ± 3% in the KA group, 93% ± 6% in the sham group, 92% ± 6% in the auricular group, and 110% ± 8% in the Zusanli group. However, no significant differences were observed among these groups (all p > 0.05; Fig. 2A and F; n = 6).

The hippocampal MCP-1 levels were 106% ± 11% in the normal group, 105% ± 9% in the KA group, 102% ± 8% in the sham group, 104% ± 10% in the auricular group, and 107% ± 12% in the Zusanli group. However, no significant differences were observed among these groups (all p > 0.05; Fig. 2A and G; n = 6).

The hippocampal CCR2 levels were 83% ± 7% in the normal group, 82% ± 8% in the KA group, 80% ± 11% in the sham group, 76% ± 9% in the auricular group, and 82% ± 8% in the Zusanli group. However, no significant differences were observed among these groups (all p > 0.05; Fig. 2A and H; n = 6).

#### IHC staining findings

**Figure 3:**
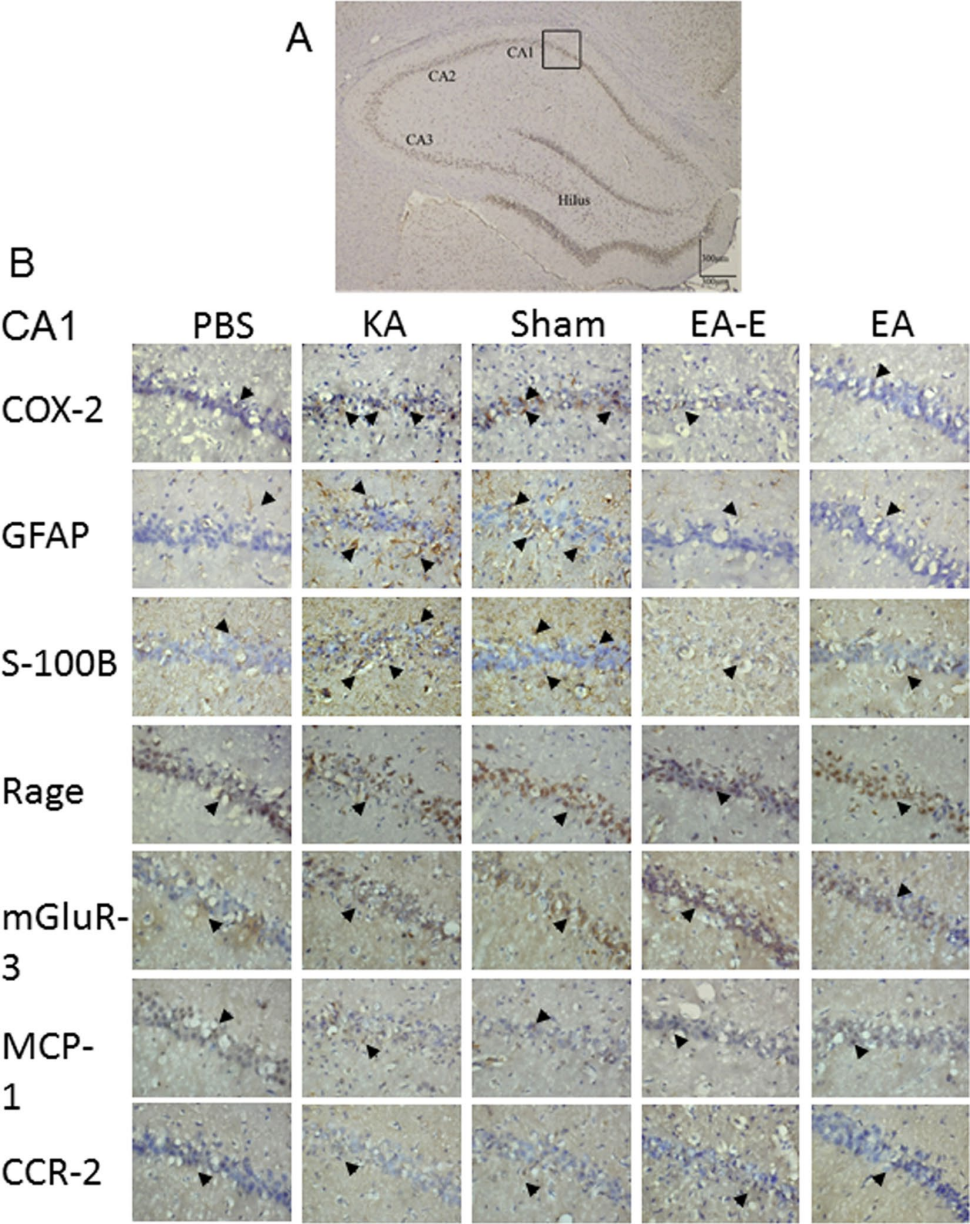
Effects of electric stimulation at the ear and electro-acupuncture at ST36–ST37 on the counts of COX-2, GFAP, S100-B, RAGE, mGluR3, MCP-1, and CCR2 immunoreactive cells in kainic acid (KA)-induced epileptic seizure rats. The CA1, CA2, CA3, and hilus regions of the hippocampus (**A**). The counts of COX-2, GFAP, and S100-B immunoreactive cells were higher in the KA group (KA) and sham group (Sham) than in the normal group (Normal), whereas these counts were decreased in the auricular group (Ear) and Zusanli group (Zusanli). The counts of RAGE, mGluR3, MCP-1, and CCR2 immunoreactive cells were similar among the normal, KA, sham, and auricular groups (**B**). Immunoreactive cell (arrowhead); B image is 400× in the CA1 region; n = 6.

The counts of the COX-2, GFAP, S100-B, RAGE. mGluR3, MCP-1, and CCR2 immunoreactive cells were assessed in the CA1, CA2, CA3, and hilus regions of the hippocampus (Fig. 3A).

The total counts of COX-2 immunoreactive cells in the CA1, CA2, CA3, and hilus regions of the hippocampus were higher in the KA group (164.8 ± 93.5) than in the Zusanli group (61.0 ± 9.0; p < 0.05; Fig. 3B; n = 6); The total counts of COX-2 immunoreactive cells were higher in the sham group (178.3 ± 79.6) than in the Zusanli and auricular (73.5 ± 29.8) groups (both p < 0.05; Fig. 3B; n = 6). The total counts of COX-2 immunoreactive cells were similar between the KA and sham groups (p > 0.05; Fig. 3B; n = 6). Because the total counts of COX-2 immunoreactive cells in the KA group were similar to those in the normal group (91.8 ± 36.9; p > 0.05), we further analyzed the individual counts in the CA1, CA2, CA3, and hilus regions. The counts of COX-2 immunoreactive cells in the CA1 region were higher in the sham group than in the normal, Zusanli, and auricular groups (all p < 0.05; Table 1; n = 6), and the counts in the KA group were higher than those in the Zusanli and auricular groups (both p < 0.05; Table 1). The counts of COX-2 immunoreactive cells in the CA2 region were higher in the sham group than in the Zusanli and auricular groups (both p < 0.05; Table 1; n = 6). The counts of COX-2 immunoreactive cells in the CA3 and hilus regions did not significantly differ among the normal, KA, sham, auricular, and Zusanli groups (all p > 0.05; Table 1). Thus, our results revealed that COX-2 increases mainly occurred in the CA1 region.

**Table 1:** Effect of electrical stimulation at the ear and electro-acupuncture at ST36–ST37 on COX-2, GFAP, and S100-B immunoreactive cells in kainic acid-induced epileptic seizure rats.

	Groups				
	Normal	KA	Sham	Ear	Zusanli
**COX-2**					
CA1	19.3 ± 13.4	42.2 ± 19.2	49.8 ± 28.2^¶^	7.8 ± 3.0*^#^	6.2 ± 4.5*^#^
CA2	8.8 ± 6.6	12.8 ± 12.6	18.2 ± 6.9	1.8 ± 1.3^#^	4.8 ± 5.5^#^
CA3	30.0 ± 13.9	48.8 ± 32.2	59.0 ± 28.8	28.2 ± 18.2	22.0 ± 5.9
Hilus	33.7 ± 11.1	61.0 ± 41.5	51.3 ± 35.2	35.7 ± 14.0	28.0 ± 9.0
**GFAP**					
CA1	22.5 ± 4.9	91.2 ± 26.6^¶^	85.2 ± 43.1^¶^	42.3 ± 21.8*^#^	36.3 ± 9.8*^#^
CA2	9.7 ± 3.9	36.7 ± 6.2^¶^	41.5 ± 30.1^¶^	13.8 ± 6.7^#^	12.3 ± 6.2^#^
CA3	18.7 ± 7.0	67.8 ± 9.6^¶^	60.2 ± 19.4^¶^	28.0 ± 9.6*^#^	25.7 ± 11.1*^#^
Hilus	65.0 ± 23.6	122.8 ± 16.6^¶^	84.3 ± 16.6*	58.8 ± 10.2*	63.3 ± 23.7*
**S100-B**					
CA1	62.7 ± 6.9	109.5 ± 11.6^¶^	111.7 ± 15.0^¶^	73.5 ± 8.7*^#^	65.7 ± 15.4*^#^
CA2	40.7 ± 6.4	48.2 ± 7.4	58.8 ± 10.7^¶^	34.3 ± 4.6*^#^	38.3 ± 10.0^#^
CA3	46.3 ± 11.7	73.8 ± 9.5^¶^	89.5 ± 11.1^¶^	52.5 ± 7.5*^#^	56.3 ± 8.8*^#^
Hilus	37.7 ± 14.8	74.7 ± 9.9^¶^	89.7 ± 14.1^¶^	45.2 ± 10.2*^#^	48.0 ± 14.6*^#^
**GFAP+S100-B**					
CA1	9.3 ± 2.1	22.0 ± 3.4^¶^	29.5 ± 4.0^¶^	40.7 ± 3.9^¶^*^#^	27.2 ± 5.6^¶^
CA2	1.5 ± 0.6	5.7 ± 1.2^¶^	8.3 ± 1.5^¶^	12.0 ± 1.2^¶^*^#^	6.3 ± 2.8^¶^
CA3	6.3 ± 1.5	19.0 ± 1.1^¶^	21.3 ± 5.2^¶^	32.3 ± 4.0^¶^*^#^	19.7 ± 4.5^¶^
Hilus	22.2 ± 1.6	37.7 ± 2.9^¶^	33.7 ± 5.9^¶^	59.7 ± 9.9^¶^*^#^	30.3 ± 5.0^¶^

Data represented as mean ± standard deviation; Normal: normal group; Sham: sham group; Ear: auricular group; Zusanli: Zusanli group; KA: kainic acid group; COX-2: cycloxygenase-2 immunoreactive cells; GFAP: glial fibrillary acidic protein immunoreactive cells; S100-B: S100-B immunoreactive cells; CA1: CA1 region of the hippocampus; CA2: CA2 region of the hippocampus; CA3: CA3 region of the hippocampus; Hilus: hilus region of the hippocampus; ^¶^p < 0.05 compared with the value of Normal; *p < 0.05 compared with the value of KA; ^#^p < 0.05 compared with the value of Sham; n = 6.

The total counts of GFAP immunoreactive cells in the CA1, CA2, CA3, and hilus regions of the hippocampus were higher in the KA (318.5 ± 39.7) and sham (271.2 ± 70.2) groups than in the normal (115.8 ± 35.9), Zusanli (137.7 ± 44.8), and auricular (143.0 ± 45.0) groups (all p < 0.05; Fig. 3B; n = 6). The total counts of GFAP immunoreactive cells in the KA and sham groups were similar (p > 0.05; Fig. 3B; n = 6). The counts of GFAP immunoreactive cells in the CA1 and CA3 regions were higher in the KA and sham groups than in the normal, auricular, and Zusanli groups (all p < 0.05; Table 1). The counts of GFAP immunoreactive cells in the CA2 region were higher in the KA group than in the normal group (p < 0.05; Table 1), and the counts in the sham group were higher than those in the normal, auricular, and Zusanli groups (all p < 0.05; Table 1). The counts of GFAP immunoreactive cells in the hilus region were higher in the KA group than in the normal, sham, auricular, and Zusanli groups (all p < 0.05; Table 1). Thus, the GFAP immunoreactive cells were distributed in the CA1, CA2, CA3, and hilus regions of the hippocampus.

The total counts of S100-B immunoreactive cells in the CA1, CA2, CA3, and hilus regions of the hippocampus were higher in the KA (306.2 ± 11.7) and sham (349.7 ± 26.1) groups than in the normal (187.3 ± 26.9), auricular (205.0 ± 25.5), and Zusanli (208.3 ± 38.6) groups (all p < 0.05; Fig. 3B; n = 6). The total counts of S100-B immunoreactive cells in the KA and sham groups were similar (p > 0.05; Fig. 3B; n = 6). The counts of S100-B immunoreactive cells in the CA1, CA3, and hilus regions of the hippocampus were higher in the KA and sham groups than in the normal, auricular, and Zusanli groups (all p < 0.05; Table 1). The counts of S100-B immunoreactive cells in the CA2 region were higher in the KA group than in the auricular group (p < 0.05; Table 1), and the counts in the sham group were higher than those in the normal, auricular, and Zusanli groups (all p < 0.05; Table 1). Thus, the S100-B immunoreactive cells were distributed in the CA1, CA2, CA3, and hilus regions of the hippocampus.

The total counts of hippocampal RAGE immunoreactive cells were 202.2 ± 109.6 in the normal group, 239.7 ± 138.0 in the KA group, 278.3 ± 104.8 in the sham group, 235.0 ± 171.3 in the auricular group, and 159.3 ± 135.4 in the Zusanli group. However, no significant difference was observed among these groups (all p > 0.05; Fig. 3B; n = 6).

The total counts of mGluR3 immunoreactive cells in the CA1, CA2, CA3, and hilus regions of the hippocampus were 192.0 ± 79.8 in the normal group, 214.7 ± 117.0 in the KA group, 185.3 ± 103.4 in the sham group, 177.7 ± 90.1 in the auricular group, and 158.0 ± 97.1 in the Zusanli group. However, no significant differences were observed among these groups (all p > 0.05; Fig. 3B; n = 6).

The total counts of MCP-1 immunoreactive cells in the CA1, CA2, CA3, and hilus regions of the hippocampus were 39.2 ± 35.3 in the normal group, 63.0 ± 59.0 in the KA group, 52.7 ± 32.1 in the sham group, 56.7 ± 27.3 in the auricular group, and 70.0 ± 59.1 in the Zusanli group. However, no significant differences were observed among these groups (all p > 0.05; Fig. 3B; n = 6).

The total counts of CCR2 immunoreactive cells in the CA1, CA2, CA3, and hilus regions of the hippocampus were 77.8 ± 34.6 in the normal group, 77.8 ± 24.2 in the KA group, 76.3 ± 48.9 in the sham group, 80.5 ± 28.8 in the auricular group, and 73.5 ± 28.0 in the Zusanli group. However, no significant differences were observed among these groups (all p > 0.05; Fig. 3B; n = 6).

### Effect of 6-week ear ES and EA at ST36–ST37 observed on double immunofluorescence analysis in KA-induced epileptic seizure rats

**Figure 4:**
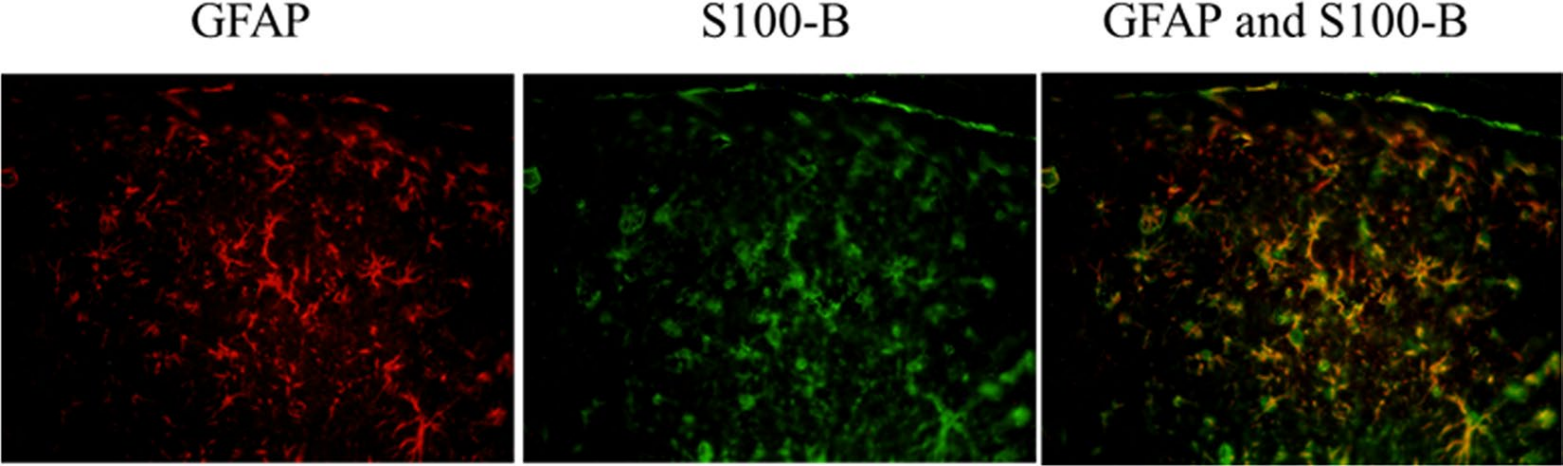
Effect of electrical stimulation at the ear and electro-acupuncture at ST36–ST37 observed on double immunofluorescence analysis in kainic acid-induced epileptic seizure rats. Double immunofluorescence analysis revealed the co-localization of GFAP (green) and S-100B (red) immunoreactive cells; the co-localization is marked with yellow (GFAP and S-100B).

The double immunofluorescence analysis of the hippocampus used IHC staining to assess GFAP and S100B. In KA-induced epileptic seizure rats, the co-localization of GFAP with S100B staining was observed in the hippocampus (Fig. 4). Significant increase of double staining of GFAP and S100B was obtained in the auricular group (Table 1).

## Discussion

Results of the present study indicated that COX-2 levels in the KA-induced epileptic seizure rats were increased, but this increase was attenuated through the 6-week application of ear ES and EA at ST36–ST37. The counts of COX-2 immunoreactive cells in the CA1 region were higher in the sham group than in the normal, auricular, and Zusanli groups, and these counts were decreased through the 6-week application of ear ES and EA at ST36–ST37. These findings suggest that COX-2 levels were increased mainly in the CA1 region of the hippocampus, but the Western blot analysis did not discriminate among the CA1, CA2, CA3, and hilus regions. Our results were consistent with the finding that dendritic excitability originates from the CA1 region of the hippocampus in KA-treated rats^22^. CA1 plays an essential role in maintaining the balance between excitation and inhibition during epileptic seizures because perisomatic inhibitory input is provided when survival pyramidal cells are preserved in the CA1 region in patients with epilepsy^23^. In patients with temporal epilepsy and pilocarpine-treated epileptic rats, axon collateral increases in the pyramidial cells, and this increase can extend to the stratum pyramidale and stratum radiatum within the CA1 region. This reorganization of hippocampal CA1 plays a critical role in epileptogenesis^24^.

COX-2 is an inflammatory mediator that produces an early inflammatory response to damage and plays a critical role in postseizure inflammation and neuronal hyperexcitablity^4^. A therapeutic strategy for drug-resistant epilepsy involves the use of COX-2 inhibitors to prevent the upregulation of seizure-induced P-glycoprotein at the blood–brain barrier^25^.

In addition, our results revealed increased GFAP levels in the KA-induced epileptic seizure rats, and these levels were decreased through ear ES. The total counts of the GFAP immunoreactive cells in the CA1, CA2, CA3 and hilus regions of the hippocampus were increased, and these counts were decreased through the 6-week application of ear ES and EA at ST36–ST37.

In the Western blot analysis, the S100-B levels did not increase in the KA-induced epileptic seizure rats. The counts of S100-B immunoreactive cells in the CA1, CA2, CA3 and hilus regions of the hippocampus increased in KA-induced epileptic seizure rats, and these counts were decreased through the 6-week application of ear ES and EA at ST36–ST37.

GFAP is an astrocyte marker, and astrocytes are a pathological hallmark of the CNS because they respond to CNS injuries through the process of astrogliosis^26^. Astrocytes modulate the release of neurotransmitters, such as glutamate uptake through the GLT transporter, and regulate the release of GABA from interneurons. The disrupted balance between glutamate and GABA levels is a key pathological mechanism in epilepsy^27^. In addition, astrocytes may play a role in amplifying, maintaining, and spreading neurogenic seizure activity^5^.

S100-B is synthesized in and secreted from astrocytes to the extracellular space^28, 29^. The serum levels of S100-B are increased in patients with mesial temporal lobe epilepsy, and serum S100-B levels are suspected to be a peripheral marker for astrocytes and brain inflammation^30^. Taken together, this suggests that most S100-B is limited within astrocytes after the 6-week application of ear ES or EA at ST3–-ST37. Accordingly, our results indicated that the counts of S100-B immunoreactive cells increased, but Western blot analysis did not reveal an increase in the S100-B levels. This finding is supported by our results indicating the co-localization of S100-B and GFAP in the double stain analysis.

In addition, both RAGE and mGluR3 levels in the Western blot study and immunoreactive cell counts in the IHC staining analysis were similar among the normal, KA, sham, auricular, and Zusanli groups. Moreover, S100-B has a dual role in intracellular and extracellular signaling. Intracellular S100-B plays a critical role in cell proliferation, migration and differentiation, and repair. Extracellular S100-B engages RAGE and has a beneficial or detrimental action depending on the protein concentration^31, 32^. High concentrations of extracellular S100-B, which result in apoptosis, can be observed in conditions such as brain trauma and inflammatory diseases. S100-B also plays a critical role in epileptogenesis^33^. mGluR3-mediates the release of S100-B from astrocytes to the extracellular space, and this extracellular S100-B modulates neuronal network activity through RAGE^34^.

The results of the present study indicated that the levels of MCP-1 and CCR2 were similar in the Western blot analysis, and the counts of MCP-1 and CCR2 immunoreactive cells in the hippocampus were also similar among the normal, KA, sham, auricular, and Zusanli groups at 6 weeks after KA-induced epileptic seizures. MCP-1 is a chemokine that plays a crucial role in the regulation of monocyte/macrophage migration and infiltration, and CCR2 is the receptor of MCP-1^11^. CCR2 has a dual proinflammatory and anti-inflammatory action^11^. MCP-1 enhances neuronal excitability through presynaptic glutamate release^35^.

The absence of increases in MCP-1 and CCR2 levels in the KA and sham groups remains unexplained. MCP-1 upregulation during seizure injury has been reported in a KA-induced status epilepticus model^36^. MCP-1 is produced by various cell types, including astrocytes, monocytes, endothelial cells, smooth muscle cells, and microglial cells^37^, and various cells may contribute to the expression of CCR2 at different time points after seizures^38^. Therefore, the relationship between the time points of MCP-1/CCR2 expression and KA-induced epileptic seizures needs further study. In addition, the limitation of the current study is that we cannot online monitor the animal behaviors during seizure during 6 weeks’ period and test actual effect of ear ES or EA.

In conclusion, the 6-week application of ear ES and EA at ST36–ST37 led to decreased COX-2 levels in the CA1 region as well as lowered astrocyte counts and S100-B levels in the hippocampus, suggesting that long-term ear ES or long-term EA at ST36–ST37 may produce an anti-inflammatory response in KA-induced epileptic seizure rats. Thus, the use of these techniques may be a strategy for the treatment of epilepsy.
